# Adiponectin in Osteoarthritis: Pathophysiology, Relationship with Obesity and Presumptive Diagnostic Biomarker Potential

**DOI:** 10.3390/diagnostics12020455

**Published:** 2022-02-10

**Authors:** Iosif Ilia, Diana Nitusca, Catalin Marian

**Affiliations:** 1 Department of Biochemistry and Pharmacology, Victor Babeş University of Medicine and Pharmacy, Pta Eftimie Murgu Nr. 2, 300041 Timişoara, Romania; iojiilia@yahoo.com (I.I.); cmarian@umft.ro (C.M.); 2 Center for Complex Networks Science, Victor Babeş University of Medicine and Pharmacy, Pta Eftimie Murgu Nr. 2, 300041 Timişoara, Romania

**Keywords:** adiponectin, osteoarthritis, obesity, diagnostic, biomarker

## Abstract

Osteoarthritis (OA) is a multifactorial, irreversible age- and obesity-induced joint degenerative disease, with an increasing incidence in developed countries. With a pathophysiology and etiology that are currently under-investigated, the only available disease-modifying treatment relies solely on total joint arthroplasty, which entails major economic burdens. Recently, the research focus has shifted towards the evaluation of metabolically active mediators secreted by the adipose tissue, which could be potential targets for a better understanding of the mechanisms involved in OA onset and development. Of note, adiponectin has drawn a great deal of attention, since it is the most abundant type of circulating adipokine and has been highly associated with OA occurrence. Thus far, studies have been controversial in establishing whether adiponectin possesses a destructive or protective role in OA development. Therefore, we critically and systematically reviewed, herein, the roles of adiponectin in the pathophysiology of OA, the link between obesity, adiponectin expression and the progression of OA, as well as its potential role as a future biomarker for a more optimized and reliable diagnosis of this degenerative disorder.

## 1. Introduction

Osteoarthritis (OA) is a very common type of age-related, injury-induced joint degenerative disorder, being undoubtedly the most common form of arthritis [1]. Novel population-based research has shown that OA has an increasing incidence and prevalence, affecting millions of individuals worldwide (incidence rising from 7.36 per 1000 persons in 2013 to 8.23 per 1000 persons in 2017) [2]. Clinically, OA is characterized by cartilage degradation of the articular joints, bone sclerosis, soft tissue damage and joint malfunction, which are processes mainly mediated by pro-inflammatory factors that maintain a permanent low-grade inflammation state of the joint, especially at the knee level [3,4]. To date, the pathophysiology and etiology of OA are not fully elucidated, and available disease-modifying therapies are scarce, with joint replacement surgery (joint arthroplasty) being the solitary end-point treatment, which is a major source of economic burden [5].

One of the primary risk factors for OA development is age; thus, the increase in global lifespan can, at least partly, explain the age-related increase in incidence (OA is more common over the age of 65). Therefore, it is one of the leading causes of chronic pain, loss of function and global disability, due to its irreversible nature [6]. Apart from age, one of the most predominant and preventable risk factors for OA development is obesity. Bodyweight is highly correlated with OA occurrence and progression, increasing both mechanical stress on the cartilage and the production of inflammatory factors arising from the adipose tissue, such as metabolically active mediators (chemokines, pro-inflammatory cytokines, and adipokines) [7,8]. Interestingly, mounting evidence of novel research corroborates the association between obesity-induced adipokine production and OA onset. Of note, adiponectin (a protein that has the following three isoforms: low-molecular-weight (LMW) trimers, medium-molecular-weight (MMW) hexamers, and high-molecular-weight (HMW) oligomers) appears to be a multifactorial key player in OA pathophysiology, and its levels can be easily detected in serum or synovial fluid sample specimens [9,10].

Functionally, adiponectin (a 244-amino-acid-long collagen-like hormone protein, also referred to as AdipoQ) is involved in the modulation of energy metabolism, such as glucose and fatty acid oxidation, insulin sensitivity, inflammation, and atherosclerosis, being the most abundant circulating adipokine secreted by the adipose tissue [11,12,13]. In addition, adiponectin has recently drawn a great deal of attention, due to its multifactorial—genetic, biochemical, and functional—associations with obesity-related OA onset. Therefore, Jiang et al. (2018) hypothesized that a single nucleotide polymorphism (rs182052) in the AdipoQ gene might be responsible for the change in susceptibility to knee OA in Chinese individuals [14]. Furthermore, from a biochemical point of view, paramount research, including case–control study designs, has found that patients with diagnosed OA (especially knee OA) had significantly higher levels of adiponectin in their samples (plasma, serum, and synovial fluid) compared to healthy controls, which also negatively correlated with obesity [15,16,17]. Additionally, three adiponectin receptors (AdipoR1, AdipoR2, and T-cadherin) have been identified in human and murine chondrocytes, with different affinities for the full-length adiponectin protein, being involved in a myriad of signaling pathways, such as peroxisome proliferator-activated receptor alpha (PPARα), 5’adenosine monophosphate-activated protein kinase (AMPK), and mitogen-activated protein kinase (MAPK) [18,19].

Thus, as a regulatory role, adiponectin appears to be involved in altering the metabolic balance of the joints via the regulation of cell growth, and the differentiation and production of matrix-degrading enzymes and other cytokines that could result in articular cartilage damage, ultimately leading to OA development [20]. High adiponectin levels were also found to be positively correlated with the Kellgren–Lawrence (KL) grading system, accounting for the X-ray examination of joint degeneration [21]. Taken together, these data highly suggest that adiponectin is not only a crucial mediator in OA pathogenesis, but could also represent a novel biomarker for reliable diagnosis. However, the findings are still controversial and contradictory, since a few studies showed no statistical differences in the adiponectin levels between OA patients and healthy subjects, while others demonstrated an opposite, protective role of adiponectin in OA progression [22,23]. Statistical data are also very limited in assessing the true diagnostic value for adiponectin in OA. Therefore, we critically and systematically reviewed, herein, the latest available literature findings on the roles of adiponectin in association with OA, for a better understanding and holistic evaluation of its involvement in OA pathophysiology and utility as a diagnostic biomarker.

## 2. Materials and Methods

### 2.1. Study Selection Process

We carried out an extensive review of the latest available literature data (up until December 2021) evaluating the roles, association, and diagnostic value of adiponectin, and its relationship with OA onset and development. Our aim was to (1) investigate the current findings regarding the pathophysiology of OA and its relationship with adiponectin, (2) review the association between obesity, high adiponectin production and OA occurrence, and (3) evaluate whether adiponectin could potentially represent a promising diagnostic biomarker for OA detection.

All studies included in this systematic review were retrieved by two independent investigators interrogating PubMed, Google Scholar, DirectScience and Web of Knowledge databases (up to 5 December 2021) using the following combination of words: osteoarthritis, knee osteoarthritis, hand osteoarthritis, hip osteoarthritis, pathogenesis, pathophysiology, association, adipokines, adiponectin, obesity, diagnostic biomarker. These medical subject heading (MeSH) terms were combined using “AND” and “OR” functions. Relevant references from included articles were also screened to identify potentially eligible studies. Included studies were published between 2008 and 2021.

Following electronic search, duplicate references were removed. Titles and abstracts were examined based on the inclusion and exclusion criteria. Two authors (D.N. and I.I.) selected the evidently relevant studies by examining the titles and abstracts, followed by full-text articles to be included. Eventual disagreements were resolved by discussion and consulting a third author (C.M.).

### 2.2. Inclusion and Exclusion Criteria

We selected articles that contained data regarding the association of adiponectin with OA and obesity-induced OA, and case–control reports evaluating the diagnostic biomarker role of adiponectin for OA detection.

Inclusion criteria were as follows: (1) studies published in peer-review journals evaluating the roles of adiponectin in OA pathophysiology; (2) studies linking obesity to adiponectin and OA; (2) case–control reports measuring adiponectin expression levels in OA patients’ samples (plasma, serum, and synovial fluid) and in healthy controls; (3) OA patients were diagnosed based on gold-standard X-ray radiography and classified based on KL grading scores; (4) articles were published in English language.

Exclusion criteria were as follows: (1) non-original papers, such as abstracts and letters to editors; (2) articles not written in English language; (3) duplicate studies; (4) articles with insufficient data for our interests.

Our study protocol was not prospectively registered.

### 2.3. Data Extraction and Characteristics of the Studies

For our third aim, we selected data regarding subject populations, sample size, specimen (plasma, serum, synovial fluid, and cartilage), OA diagnosis, OA stage, expression level of adiponectin, detection method, correlations with other parameters and proposed effects. Given that the majority of the included eligible articles did not contain sufficient data for a meta-analysis, the present report is a structured review of the presently available data.

In our structured review, we only included articles with data regarding the association of adiponectin with OA onset and progression, obesity-induced OA, and the link with adiponectin levels, and data regarding adiponectin expression levels in OA patients’ samples and healthy controls.

**Figure 1:**
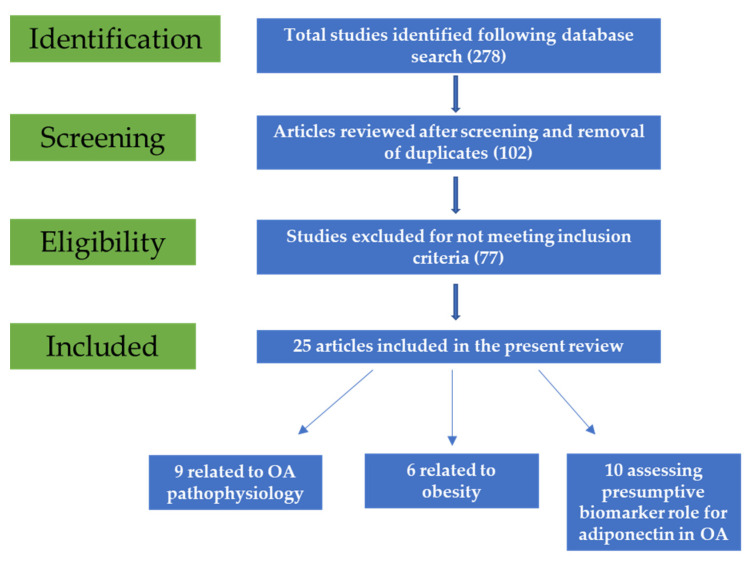
Flow diagram of the study selection process.

Following initial search, 278 potentially relevant articles were retrieved. After title and abstract screening, 102 studies were included. Duplicate reports were also removed. Following full-text reading, 77 articles were removed as they did not meet the inclusion criteria, and, finally, 25 were included in the present review (Figure 1).

## 3. Results

### 3.1. Adiponectin in Relationship with OA Development

Emerging research has displayed both the pro-inflammatory and catabolic roles of adiponectin in OA pathogenesis. It appears that, at the AdipoR1 level, from both the chondrocytes and the osteoarthritis synovial fibroblasts (OASFs), high adiponectin levels stimulate via the AMPK and nuclear factor (NF-κB) signaling pathways for the release of pro-inflammatory interleukins (such as IL-6), matrix metalloproteases (MMP-1 and -3), and the production of inducible nitric oxide synthase (iNOS), generating pain, inflammation, and matrix degradation [24,25]. The production of IL-6 seemed to be time and concentration-dependent, and successfully inhibited by transfection with small interference RNA (siRNA) and/or pre-treatment with AMPK pathway inhibitors (AraA and compound C). Mechanistically, adiponectin stimulated phosphorylation and increased the kinase activity of AMPK and p38, p65 and p70 translocation from the cytoplasm to the nucleus, and mediated an increase in κB luciferase activity, among other effects [24].

**Figure 2:**
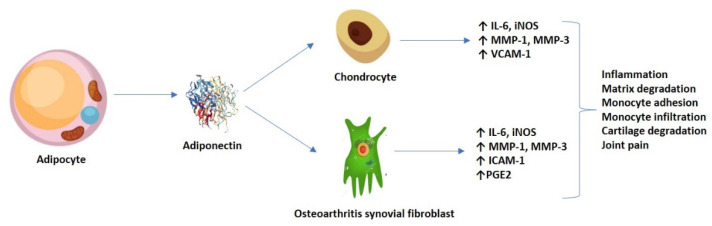
Adiponectin-induced expression of molecules in both chondrocytes and OASFs, and their resulting effect.

In addition, Chen et al. (2014) previously demonstrated that adiponectin might increase ICAM-1 expression via several signaling pathways, thus promoting monocyte adhesion and infiltration in OA progression. Adiponectin stimulation in OASFs (monocyte THP-1 cell line) promoted the phosphorylation of two AMPK upstream activators–liver kinase B1 (LKB1) and calmodulin-dependent protein kinase II (CaMKII)—resulting in downstream factors (c-Jun and AP-1) binding to the ICAM-1 promoter [26]. Nonetheless, the persistent articular cartilage damage could also be maintained by adiponectin-induced expression of vascular cell adhesion molecule 1 (VCAM-1) in both human and murine chondrocytes [27]. Furthermore, it is hypothesized that adiponectin induced the production of inflammatory prostaglandin E2 (PGE2) in OASFs in a concentration-dependent fashion, which was relieved by treatment with non-steroidal anti-inflammatory drugs (NSAIDs) and cyclooxygenase-2 (COX-2)-selective inhibitors [28]. Figure 2 shows an overview of the adiponectin-induced expression of various molecules in chondrocytes and OASFs.

On the contrary, other reports show that adiponectin might possess protective and anti-inflammatory roles, preventing cartilage damage and degradation. Treatment with adiponectin in chondrocyte cell lines elevated the expression of the tissue inhibitor for MMP-2 (TIMP-2) and decreased gene expression for IL-1β-induced MMP-13 [29]. Moreover, Hu et al. (2017) demonstrated that adiponectin underwent autophagy that was possibly associated with the AMPK/mTOR signaling pathway in H_2_O_2_-induced apoptosis, thus having a protective role in chondrocytes [30]. Furthermore, patients displaying higher adiponectin levels in their serum seem to have a longer resilience of joint replacement, and decreased serum adiponectin is correlated with loosening at the 10-year follow-up for joint arthroplasty. It appears that adiponectin regulates both osteoblast and osteoclast activity, by increasing osteogenic markers, such as alkaline phosphatase (ALP) and osteocalcin, and inhibiting phosphatase and tensin homolog (PTEN) and osteoclastic differentiation [31]. Additional reports linked adiponectin to bone marrow stem cell differentiation through activation of the Wnt/β-catenin signaling pathways [32]. Table 1 summarizes the main findings on adiponectin and its relationship with OA.

### 3.2. The Link between Adiponectin and Obesity-Induced OA

Obesity is known to be a condition with excessive or abnormal fat accumulation, a body mass index (BMI) value over 30 kg/m^2^, and permanent low-grade inflammation, which greatly increases the likelihood of developing OA, especially at the knee level. Research efforts are undertaken in order to gain insights into the link between OA and obesity, with the long-term goal of developing prevention and focused intervention strategies. It is becoming increasingly clear that adipose tissue is not only an area for storing passive energy, but also a highly active metabolic organ that is capable of secreting a myriad of active molecules, including adiponectin. However, not all individuals with knee OA are obese and vice versa, likely due to the relative distribution of adipose tissue and its contribution to muscle strength. Therefore, obesity is considered a comorbidity and a major risk factor (but not an obligatory component) for OA, together with other metabolic, cardiovascular, and respiratory diseases [33].

There are two distinct mechanisms by which obesity might contribute to OA onset; one of which is purely mechanical, in that additional mass increases wear and joint load, resulting in degenerative changes over time. Cartilage damage and permanent breakdown occur at higher rates than articular cartilage production in obese individuals. Interventions, such as weight loss or infrapatellar fat pad (IFP) resection, have been shown to improve OA severity and reduce mechanical loading around the knee joint [34]. The other mechanism involves the capacity of the adipose tissue to promote and sustain local and systemic inflammation, by releasing a wide spectrum of pro-inflammatory active agents into circulation, including adipokines, such as adiponectin.

It has been previously demonstrated that excess fat secretes more inflammatory cytokines into circulation, as higher levels of inflammatory proteins and adipokines (including adiponectin) are observed in obese individuals. In addition, there is cross-talk between macrophages and adipocytes, which results in adipose-associated inflammatory features. The M1 phenotype, characteristic for obesity, is known to produce higher levels of adipokines, and, in turn, adiponectin (and leptin) is capable of regulating obesity-induced inflammation in OA, by modulating immune activity [35,36]. Adiponectin employs its immunomodulatory properties by suppressing the production of TNFα in macrophages, arresting phagocytosis, and by the differentiation of mononuclear precursors.

Furthermore, adiponectin inhibits the generation of foam cells and the differentiation of macrophages from the active M2 phenotype (anti-inflammatory, characteristic for non-obese individuals) to the classic activated M1 phenotype (pro-inflammatory, characteristic for obesity). It is believed that adiponectin is involved in this process not only because it decreases pro-inflammatory cytokine levels (TNFα), but also because it promotes M2 macrophage differentiation, proliferation, and expression by up-regulating anti-inflammatory M2 markers [37,38]. In addition, some studies showed that the body weight values were inversely correlated with adiponectin concentrations, but the findings remain disputable and contradictory in establishing the exact role of adiponectin in obesity-induced OA pathogenesis.

### 3.3. Can Adiponectin Represent a Future Diagnostic Biomarker for OA?

Emerging case–control reports demonstrate that, generally, adiponectin levels are higher in OA patients’ samples compared to healthy controls, although the comparison did not meet statistical significance in all cases. Tootsi et al. (2016) found that serum adiponectin levels were significantly higher in the group of patients relative to the non-OA subjects, and that OA grade was associated with MMP-3 and leptin levels. In addition, the authors have linked OA severity with the expression of adipokines, and proved that the concentration of adiponectin was positively correlated with the augmentation index of arterial stiffness [39]. This corroborated a previously published study conducted by Koskinen et al. (2011) on 35 male OA patients undergoing total knee replacement surgery, who found that plasma and cartilage adiponectin levels were significantly higher for the subgroup of OA patients in the most severe form (Ahlbäck grades 4 and 5), and positively correlated with MMP-3 and other OA biomarkers, such as IL-6, NO, and MMP-1, thus suggesting a possible implication of adiponectin in cartilage destruction [40]. Adiponectin was found to have higher concentrations in OA patients’ samples vs. controls in additional reports as well, reinforcing the previous findings; furthermore, adiponectin and leptin were associated with female gender, body mass index, and synovial inflammation, suggesting a potential role for adiponectin in the vigorous inflammatory component of OA [16,41]. Adiponectin levels from the serum were also negatively associated with bone mineral density (BMD) at the total femur and femoral shaft, and positively correlated with degradation markers of the cartilage matrix in synovial fluid samples, such as aggrecan AGG1 and AGG2, suggesting another potential role for adiponectin in regulating the degradation of the cartilage matrix in OA progression [42,43].

However, another study discovered higher adiponectin levels in the serum samples of OA patients compared to healthy subjects, but the difference did not reach statistical significance following the adjustment of variables (age, gender, and weight). However, it seems that adiponectin levels were positively associated with KL scores, thus suggesting a potential disease severity biomarker role for future clinical practice [21]. On the other hand, one study did not find any statistical significance in the differential expression of adiponectin in OA patients vs. controls, but discovered, instead, that adiponectin levels were remarkably higher in the plasma relative to the paired synovial fluid (*p* < 0.001), and negatively correlated with the OA severity measured by the KL grading system, suggesting a protective role for adiponectin in OA [22]. Similar results were found by Zheng et al. (2016) in a cross-sectional study comprising a total of 205 patients, in which serum adiponectin, but not resistin or leptin, was found to be significantly associated with reduced OA severity, even after variables, such as age, sex, weight, and disease duration, were adjusted for, once more proposing a potentially protective effect for this adipokine [23].

Nonetheless, the aforementioned studies focus mostly on knee OA, since it is not only one of the most studied types of OA (from the joint-affected point of view), but also highly connects with obesity-induced OA (mainly due to the mechanical load, which was mentioned in a section above). In addition, other types of OA (hip and hand) [39,44,45,46,47,48,49], together with knee OA, are presented in Table 2.

## 4. Discussion and Future Perspectives

Thus far, adiponectin has been highly associated with different forms and stages of OA; however, its exact role in OA pathogenesis remains unclear, since study reports reveal both destructive and protective properties. Contradictory findings might also suggest a dual or more complex role of adiponectin in OA, or could have simply arisen due to large differences in study designs, and/or the methodology of choice. Although accumulating in vitro and in vivo evidences have underpinned the metabolic relationship between OA and adiponectin, the exact and direct mechanism of adipokines in OA onset and development remains largely unknown and understudied. It is, therefore, especially important that future case–control and longitudinal studies introduce, in their analysis, a pertinent cohort size, and that a consensus is applied to ensure that they have a uniformized methodology approach.

Furthermore, the lack of appropriate diagnostic and classification tools introduces considerable heterogeneity to the sample populations, and differences in the baseline demographics could introduce a certain amount of bias that makes duplication of the results a difficult task. The major drawback of the current diagnostic tools for OA (mainly X-ray radiography) is that they usually detect changes in advanced or end-stages, while OA undergoes a silent, gradual process of progression. For this reason, the aforementioned reports summarized in the present review suggest that adiponectin might possess a dual role in OA pathophysiology, and opens avenues for future research in the diagnostic areas.

However, the major limitation of our study arises from the fact that the majority of the studies assessing a biomarker role for adiponectin in diagnosing OA do not provide sufficient relevant statistical data regarding fold changes or area under the curve (AUC) values, and, therefore, the biomarker status remains only at a level of hypothesis, to date. Moreover, conflicting results across studies could also arise, due to the existence of isoforms of adiponectin with different molecular weights, which are not usually mentioned within methodologies. Reviewing the role of adiponectin in OA diagnosis, and taking this aspect into account, remains, thus, an unfeasible assignment momentarily.

Taken together, the tight relationship between obesity-induced OA and adiponectin opens novel avenues for study, and strongly advocates that OA is a multifactorial, systemic disease, triggered by various processes, such as metabolic imbalance and immune-mediated inflammation, involving different tissues and organs. It is ubiquitously accepted that excess adipose tissue is not only a source for joint load, but also a portal for a plethora of inflammatory cytokines, including adipokines (adiponectin). Understanding their exact pathophysiological mechanism is crucial for the development of precise and reliable biomarkers, as well as modern therapeutic agents that could decrease both the economic burden of joint replacement surgeries and the morbidity of this degenerative disorder.

## Figures and Tables

**Table 1:** Adiponectin-induced effects and the repercussions for OA development.

Cell Type	Induced Effect	Signalling Pathway	Effects for OA	Reference
Chondrocyte & OASFs	↑ IL-6, MMP-1, MMP-3, iNOS	NF-κB AMPK	Destructive: Pain Inflammation Matrix degradation	[24,25]
OASFs	↑ ICAM-1	LKB1/CaMKII AMPK AP-1	Destructive: Monocyte adhesion and infiltration	[26]
Chondrocyte	↑ VCAM-1	JAK2 PI3K AMPK	Destructive: Cartilage degradation Inflammation of joints	[27]
OASFs	↑ PGE-2	-	Destructive: Inflammation	[28]
Chondrocyte	↑ TIMP-2 ↓ MMP-13 (IL-1beta-induced)	-	Protective: Preventing cartilage destruction Anti-inflammatory	[29]
Chondrocyte	↑ Bcl-2, LC3B ↓ Bax, P62	AMPK/mTOR	Protective: Antiapoptotic	[30]
Human bone marrow-derived stromal cells (hBMSC)	↑ osteocalcin, alkaline phosphatase, runt-related transcription factor-2 ↓ PTEN	-	Protective: Enhanced osteogenic differentiation Therapeutic agent in tissue and bone regeneration	[31]
Bone mesenchymal stem cell (BMSC)	↑ β-catenin, cyclinD1	Wnt/β-catenin	Protective: Bone marrow stem cells differentiation	[32]

**Table 2:** Summary of main findings regarding adiponectin expression levels and suggested effects.

Subject Population	Sample Specimen	OA Diagnosis	Adiponectin Levels	Correlations	Reference
60 patients with knee OA 25 healthy controls	Serum	Radiological evaluation (KL criteria)	Increased (not statistically significant after adjustment of variables)	KL scores (+)	[21]
76 patients with knee OA 24 healthy controls	Plasma Synovial fluid	Radiological evaluation (KL criteria)	Increased (not significantly)	OA severity (−) KL score (−)	[22]
205 patients with knee OA	Serum	Radiological evaluation (KL criteria)	-	Reduced radiographic OA severity (−)	[23]
70 end-stage knee and hip OA patients 70 healthy controls	Serum	Radiological evaluation (KL criteria)	-	Augmentation index (+) Large artery elasticity index (−)	[39]
35 knee OA patients	Plasma Cartilage	Radiological evaluation (preoperative Ahlbäck classification)	Increased in Ahlbäck grades 4 and 5	MMP-3 (+) COMP (+)	[40]
172 patients with knee OA 132 healthy controls	Serum	Radiological evaluation (KL criteria)	Increased	Female gender (+) Body mass index (+)	[41]
164 knee OA patients	Serum	Radiological evaluation (KL criteria)	-	Bone mineral density (BMD) at total femur and femoral shaft (−)	[42]
30 knee OA patients	Synovial fluid Plasma	Radiological evaluation (KL criteria)	Decreased (in synovial fluid compared to paired plasma)	AGG-1 (+) AGG-2 (+)	[43]
2402 knee and hand OA patients	Serum	X-rays using a semi-quantitative grading system	Increased	Total osteophyte score (+) Joint space narrowing (JSN) scores (+) Higher total radiographic scores; only in knee joint, but not in the hand joint	[44]
6408 knee and hand OA patients	Serum	American College of Rheumatology criteria	-	Adiponectin levels were not associated with osteoarthritis	[45]
164 patients with hand OA	Serum	KL score ≥ 2 in at least two hand joints	-	Patients in the two highest tertiles of adiponectin had a decreased risk for hand OA progression	[46]
44 patients with hand OA	Serum	Radiological evaluation (KL criteria)	-	Adipokine concentrations were not associated with hand OA radiographic severity	[47]
94 patients with hand OA and 21 healthy controls	Serum	Radiological evaluation (KL criteria)	No significant differences among groups	-	[48]
112 patients with hip OA and 94 with knee OA	Synovial fluid	Radiological evaluation (KL criteria)	Increased in hip OA compared to knee OA	Connection between intra-articular concentrations of several adipokines	[49]
